# Gene expression profiling identifies ferroptosis-related genes and pathways in human colon cancers cell lines

**DOI:** 10.3389/fmolb.2025.1680206

**Published:** 2026-01-20

**Authors:** M. Balik-Meisner, D. Phadke, D. Mav, R. Shah, K. R. Shockley, Carri Murphy, Erik J. Tokar, Birandra K. Sinha

**Affiliations:** 1 Sciome LLC, Durham, NC, United States; 2 Biostatistics and Computational Biology Branch, Division of Intramural Research, National Institutes of Environmental Health Sciences, NIH, Durham, NC, United States; 3 Mechanistic Toxicology Branch, Division of Translational Toxicology, National Institutes of Environmental Health Sciences, NIH, Durham, NC, United States

**Keywords:** colon cancer, erastin, ferroptosis, biomarker genes, pathway analysis

## Abstract

**Introduction:**

Colorectal cancer (CRC) is the third most diagnosed cancer worldwide and the second leading cause of cancer-related deaths. A major challenge in CRC treatment is drug resistance, which limits the efficacy of conventional therapies. Ferroptosis, an iron-dependent form of regulated cell death driven by the accumulation of reactive oxygen species (ROS), has emerged as a promising therapeutic strategy. Erastin (ER), a small-molecule compound, induces ferroptosis through ROS accumulation.

**Methods:**

We performed microarray gene expression analysis on two CRC cell lines, HCT116 and HT-29, to examine the transcriptional response to ER exposure and identify differentially expressed genes and pathways involved in ER-induced ferroptosis.

**Results:**

Our gene expression analysis revealed distinct transcriptional profiles between the two cell lines, and 26 transcripts commonly enriched in response to ER treatment were identified in both HCT116 and HT-29 cells. Notably, several of these genes—including *ASNS, PCK2, CHAC1, and DDIT4*—were significantly enriched, suggesting a conserved ferroptotic response. The induction of these genes was further confirmed in an additional CRC cell line, DLD-1. Interestingly, *SOD1* and *NQO1* genes, involved in oxidative stress response, were significantly upregulated by ER in HCT116 cells.

**Conclusion:**

Our findings highlight *ASNS, CHAC1, PCK2, DDIT4, and ATF3/4* as potential biomarkers for ferroptosis in CRC. Monitoring the expression of these genes may help identify patients who are responsive to ferroptosis inducers and facilitate the development of personalized treatment strategies.

## Introduction

Cancer is one of the leading causes of death worldwide. Colorectal cancer (CRC) is the third most prevalent cancer around the world, with increasing morbidity and mortality every year (Biller and Schrag, 2021; Bray et al., 2018). The incidence of CRC has been rising (Sung et al., 2021) and according to the International Agency for Research on Cancer (IARC) there were roughly 200,000 new cases of CRC and 1.0 million deaths worldwide in 2020. CRC accounted for 10% of overall cancer incidence (Sung et al., 2021). Currently, the treatment of CRC mainly consists of surgery, targeted therapy, radiotherapy, and chemotherapy (Dekker et al., 2019). Chemotherapy is one of the most used therapies for patients with advanced CRC and usually provided following surgery as adjuvant therapy. While chemotherapy has improved the overall survival of patients with advanced CRC in the past decades, many CRC patients develop drug resistance with poor survival (Wang et al., 2022). Development of chemotherapy resistance, including multidrug resistance (MDR) results in therapy failure in human CRC and other cancers in the clinic. Understanding mechanisms of drug resistance and overcoming this resistance has become a clinical priority. The development of newer drugs, selectively effective against CRC, are urgently needed.

Ferroptosis is a distinct form of regulated cell death involving cellular lipid peroxidation and iron, and it has potential therapeutic implications in cancer treatment (Dixon et al., 2014; Yang and Stockwell, 2016; Singh et al., 2025). Ferroptosis is considered a form of caspase-independent programmed cell death which relies on the mitochondrial and membrane-involved accumulation of reactive oxygen species and lipid peroxides leading to non-caspase-mediated cell death (Dixon et al., 2012). In comparison to conventional cell death forms, iron-dependent lipid peroxidation is considered a potential avenue for targeting cancer cells that display resistance to conventional chemotherapies (Chen et al., 2015; Zhou et al., 2019; Perera et al., 2025). Among various components of metabolic heterogeneity in cancer cells, iron-catalyzed pathways in lipid metabolism are more abundant in cancer cells. Ferroptosis inducers, like Erastin (ER) and RSL3, have shown promise in sensitizing resistant tumor cells to various chemotherapeutics agents (Zhou et al., 2019; Brown et al., 2024; Perera et al., 2025) and have emerged as promising candidates for addressing MDR. Cancer cells are more vulnerable to iron toxicity due to high dependency on iron metabolism than noncancerous cells, making cancer cells more vulnerable to ferroptosis. Combining ferroptosis inducers with chemotherapeutics offers a novel strategy to enhance treatment efficacy and overcome resistance.

Studies have shown that the CRC cell lines, HT-29 and HCT116, undergo ferroptosis (Wang et al., 2022; Sinha et al., 2023; Yan et al., 2023; Wu et al., 2023). Our recent study has indicated that ER and RSL3 act synergistically with NCX4040, a non-steroidal drug, to induce ferroptotic cell death in both HT-29 and HCT116 cells (Sinha et al., 2023). Pathways leading to ferroptosis are complicated and little is known about specific genes involved in inducing ferroptosis in CRC. Because no single cell line fully captures the complexity of colorectal cancer (CRC)—including tumor microenvironment, immune interactions, and heterogeneity—HCT116 and HT-29 represent two highly distinct CRC subtypes: MSI-high/KRAS-mutant (HCT116) and MSS/BRAF-mutant (HT-29). Together, they provide a broader view of CRC heterogeneity. Both are among the most cited and validated CRC models, with extensive omics data. Additionally, we have a long-standing experience working with them, including recent metabolomic studies with ER. We have, therefore, utilized human colon cancer HT-29 and HCT116 cell lines to examine the effects of Erastin short (4 h) and long (24 h) exposure on global gene expression patterns in using gene expression microarray analysis to identify molecular pathways involved in ER-induced ferroptosis in CRC.

## Methods

Chemicals: Erastin was purchased from Sigma Chemicals (St. Louis, MO, United States) and was dissolved in DMSO. Stock solutions were stored at −80 °C. Fresh drug solutions, prepared from the stock solutions, were used in all experiments.

### Cell culture and drug treatment

Authenticated human colon tumor cells, HT-29 cells, HCT116 cells and DLD-1were obtained from ATCC (Manassas, VA, United States) and were grown in Phenol Red-free RPMI 1640 media supplemented with 10% fetal bovine serum and antibiotics. Tumor cells were routinely used for 20–25 passages, after which the cells were discarded, and a new cell culture was started from the frozen stock.

Three independent experiments of exponentially growing cells (0.5–1.0^6^ cells, 65%–70% confluency) were left untreated or treated with ER (2.5 µM) for 4 h, and 24 h, and then washed twice with ice-cold PBS (pH 7.4). For the gene profiling studies presented here, we used 2.5 μM ER at shorter time points, consistent with our earlier metabolomic work (Sinha et al., 2023). This concentration was selected to minimize cytotoxicity: at 4 h, ER showed no measurable toxicity in these cells and even at later points induced only modest cell death (<20%). Total RNA was extracted with TRIzol (Ambion, Life Technologies, Grand Island, NY, United States) and purified using RNeasy mini kit columns (Qiagen, Valencia, CA, United States). The purity of RNA was assessed with a NanoDrop spectrophotometer and the 260/280 ratio determination.

### Global gene expression and data acquisition

A gene array expression analysis was conducted using Affymetrix Human Whole Transcriptome 2.0 arrays (Affymetrix, Santa Clara, CA). One hundred nanograms (100 ng) of total RNA were amplified and labeled as directed using the Affymetrix WT Plus Reagent Kit (WT Plus Kit). Five and one-half micrograms (5.5 μg) of amplified biotin-cDNAs were fragmented and hybridized to each array for 16 h at 45 °C in a rotating hybridization oven Array slides were stained with streptavidin/phycoerythrin utilizing a double-antibody staining procedure and then washed for antibody amplification per the GeneChip Hybridization, Wash and Stain Kit and user manual following protocol FS450-0001. Arrays were scanned in an Affymetrix Scanner 3000 and data was obtained using TAC Software.

The data is available in the Gene Expression Omnibus under GEO accession GSE299397 (https://www.ncbi.nlm.nih.gov/geo/query/acc.cgi?acc=GSE299397).

### Normalization

The raw probe-set level signal from CEL files were extracted for ∼1.3 million probe-sets. The *oligo* R package (Carvalho and Irizarry, 2010) was utilized to perform Robust Multiarray Average (RMA) normalization of the probe-set level signal. Normalization was performed separately for each cell line. Mean summarization was utilized to derive expression signal for ∼138K transcripts, and subsequently for ∼38K unique NCBI curated genes. Log2 transformation was applied to the normalized signal.

### Outlier detection

The systematic quality assessment of the samples was performed via principal component analysis, hierarchical clustering, and inter-replicate correlation analysis. These analyses and resulting plots were used to detect potential outliers. No samples were determined to be outliers.

### Gene and pathway analysis

Differentially expressed transcript (DET) analysis was performed using Student’s t-test and permutation-based p-value computation. DETs were detected using balanced criteria (foldchange ≥1.2 and unadjusted p-value ≤0.005).

Transcripts that passed the DET criteria were used for pathway analysis. Differentially enriched pathways (DEPs) with an overrepresentation of DETs were detected using customized functional analysis approach via normalized enrichment score (NES) and Fisher’s exact test p-value. DEP analysis was performed on the Molecular Signature Database (MSigDB v7.5.1) (Liberzon et al., 2011; Liberzon et al., 2015) on a total of 2,834 pathways that had a minimum of five genes present on the microarray. The set of pathways included 29 C2-CP pathways, 292 BIOCARTA pathways, 186 KEGG pathways, 1613 REACTOME pathways, 664 WikiPathways, and 50 Hallmark pathways. Significance criteria for DEPs required NES ≥2 and Benjamini & Hochberg adjusted p-value ≤0.05.

### RT-PCR studies

The expression levels of selected transcripts were confirmed by reverse transcription polymerase chain reaction (RT-PCR) using absolute SYBR green ROX Mix (ThermoFisher Scientific, Rochester, NY, United States) as previously described (Sinha et al., 2020). Data were analyzed using the ΔΔCt method of relative quantification and to determine transcript levels, cycle times (Ct) were compared from the same sample and were normalized to β-actin from the same sample. Primers (Supplementary Table 1) for the selected genes were purchased from Origene (Gaithersburg, MD, United States). Complementary DNA was transcribed from the purified RNA using the Bio-Rad iScript cDNA Synthesis Kit. Using the cDNA, fast SYBR green master mix and primers, the RT-PCR was performed on the Quant Studio 7 Flex to determine the expression levels of selected genes.

All experiments were conducted in triplicates (n = 3) and results are expressed as mean ± SEM. Significance was assessed using an unpaired Student’s t-test and considered significant when p < 0.05.

## Results

**FIGURE 1:**
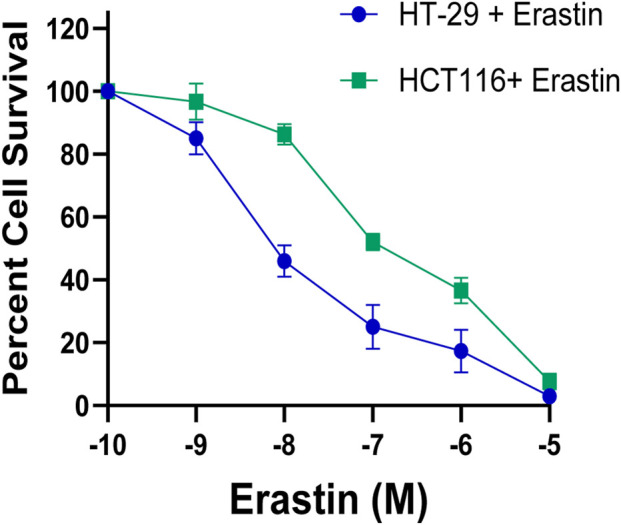
Cytotoxicity of Erastin in HT-29 and HCT116 cells as determined by CellTiterGlO (Promega) cytotoxicity assay following 72 h ER treatment.

Our previous studies have shown that ER induces ferroptosis in several human tumor cell lines, including CRC at micromolecular (1–5 µM) concentrations (Sinha et al., 2023; Perera et al., 2025). Furthermore, several investigators have also found that CRC undergo ER-mediated ferroptosis (Wu et al., 2023). In this study, we found ER to be significantly cytotoxic to both HT-29 and HCT116 cells (Figure 1). Interestingly, ER was less cytotoxic to HCT116 cells than HT-29 cells. DLD-1 cells had similar cytotoxic profiles as HCT116 cells (data not shown). While the exact mechanism is not clear, the response to ER can vary across different colon cancer cell lines. Different CRC have been shown to have different sensitivity to ER (Hu et al., 2022; Zheng et al., 2023) due to either GPX4 levels or tumor heterogeneity where different clones may have different mechanisms for resisting ferroptosis-induced cell death.

**FIGURE 2:**
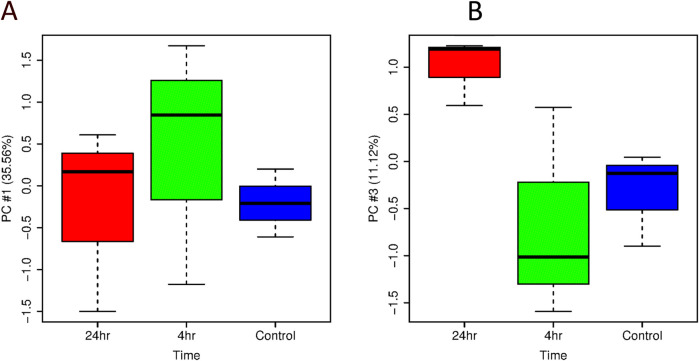
Boxplots of **(A)** PC#1 and **(B)** PC#2 from the PCA analysis of all HT-29 samples broken down by time (control, treated with ER at 4 h, treated with ER at 24 h).

**FIGURE 3:**
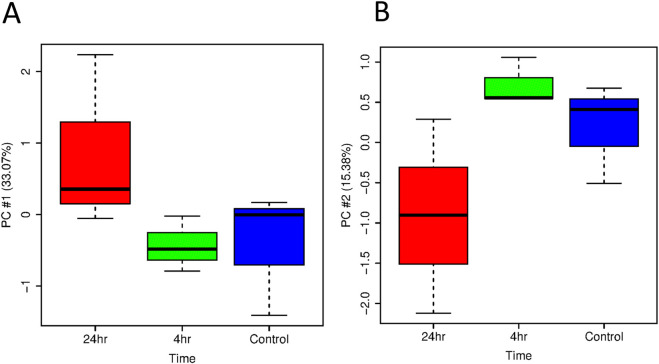
Boxplots of **(A)** PC#1 and **(B)** PC#2 from the PCA analysis of all HCT116 samples broken down by time (control, treated with ER at 4 h, treated with ER at 24 h).

Principal Component Analysis (PCA) of transcripts following ER treatment at 4 h and 24 h are shown in Figures 2, 3 using boxplots to observe the distribution of individual scores on each principal component. For HT-29 cells, there is not an obvious time effect based on PC#1 and PC#2 (not shown, captures 13% variability). 24 h samples do not differ much from Control and 4 h samples until PC#3 which indicates clustering of samples at both 4 h and 24 h (Figure 2). For HCT116 cell line, there is an observable time effect and the 24 h samples separate from Control and 4 h samples in clustering and on PC#1 and PC#2.

### Analysis of overlay of DETs in HT-29 and HCT116 cells

**FIGURE 4:**
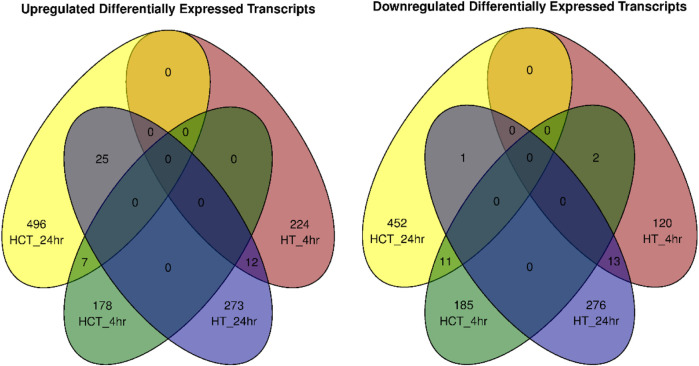
Venn diagrams of overlay of differentially expressed transcripts (DETs) at 0, 4 and 24 following ER treatment of HT-29 and HCT116 cells.

Venn diagrams of differentially expressed transcripts (DETs) showed significant number of genes were either upregulated or downregulated at early time points (4 h) in either HT-29 or HCT116 cells, however very few transcripts were commonly regulated in both cell types (Figure 4). Many transcripts, common to both cell lines, however, were differentially expressed at 24 h of ER treatment. Analysis of DETs revealed distinct transcriptional responses across the different treatment conditions (HCT116 and HT-29) and time points (4 h and 24 h). For upregulated transcripts, the majority of DETs were unique to each condition, with minimal overlap observed between groups. No transcripts were commonly upregulated across all four conditions, and only minor overlaps were noted between related groups (e.g., 25 transcripts between HCT116 24 h and HT-29 4 h). Similarly, downregulated transcripts exhibited highly condition-specific patterns. Most downregulated transcripts were unique to each condition, with extremely limited overlap between groups (1–13 transcripts shared between pairs) and no transcripts were commonly downregulated across all conditions. Together, these findings suggest the specificity and dynamic nature of gene expression regulation in response to treatment and time course.

Because there were only two DETs changed at 4 h of ER treatment in both cell types, we compared common DETs at 24 h in these cells (Supplementary Table 2). We found that 26 common transcripts were expressed in both HT-29 and HCT116 cells. Several of these transcripts are associated with genes that have been shown to be expressed during ferroptosis, e.g., *ASNS, CHAC1, DDIT4 and FYN* (Sinha et al., 2023; Gan et al., 2024; Zhou et al., 2021; Qi et al., 2025). Interestingly, ASNS, CHAC1, PCK2 and DDTI4 transcripts were noticeably highly expressed in both cell lines, indicating possible similar responses to ER treatment.

**FIGURE 5:**
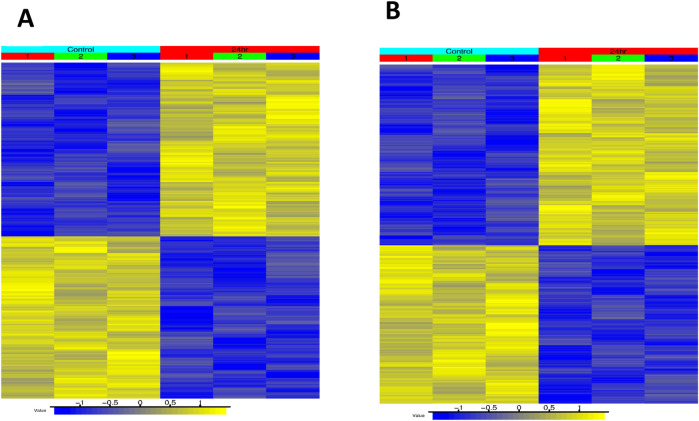
Heat map showing hierarchical clustering DETs of HT-29 [**(A)** 600 DET’s] and HCT116 [**(B)** 992 DET’s] following treatment with Erastin at 24 h. Yellow represents upregulated DET’s, and blue indicates downregulated DET’s.

Figure 5 shows clustering of DETs in HT-29 and HCT116 cells following ER treatment. The expression pattern shows a clear time-dependency of ER effects and in both cell lines, 24 h treatment elicited largest numbers DETs/genes regulated by ER. Further, the clustering pattern shows a clear difference in gene regulation in HT-29 and HCT116 cell types.

### Pathways analysis

Several differentially expressed pathways were identified in HT-29 and HCT116 cells following ER treatment (Supplementary Table 3). At 4 h ER treatment, one pathway was identified in HT-29 and no pathways in HCT116 cells. In contrast, several pathways could be identified in both cell lines at 24 h: more in HT-29 than HCT116. These findings would suggest that ER does not significantly induce early response in these cells and that HT-29 cells are more sensitive to ER-dependent late responses than HCT116 cells. Additionally, this may indicate cell line-specific sensitivity, pathway activation, or stress responses.

Furthermore, pathway analysis indicated that several pathways were common to both cell lines in response to 24 h ER treatment (Supplementary Table 4).

**FIGURE 6:**
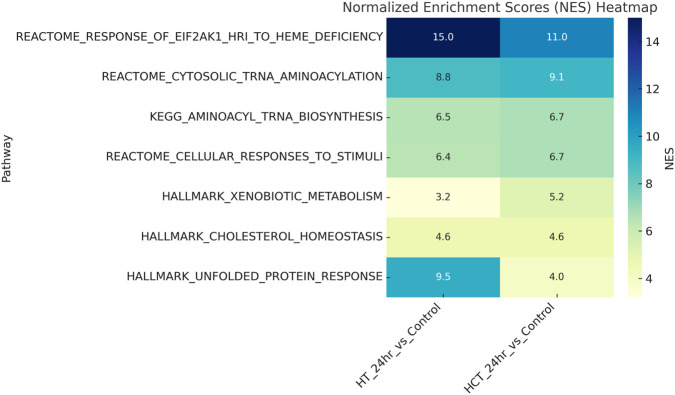
A diagrammatic presentation of common pathways modulated in both HT-29 and HCT116 cells by Erastin at 24 h exposure.

Our data indicate that several important biological pathways are modulated by ER treatment in both HT-29 and HCT116 cells. This suggests a strong stress response activation, likely due to heme or iron metabolism perturbation by ER in both cell types as they are highly enriched in both HT-29 and HCT116 cells (NES = 14.51 and 11.17, respectively) with upregulated genes. Second, Unfolded Protein Response (UPR) is also highly significant in both HT-29 and HCT116 cells, (NES 9.45 and 3.97, respectively)- six of eight significant DETs in Hallmark UPR in HT-29, all 5 UPR DETs in HCT116 are upregulated, indicating endoplasmic reticulum stress, which usually results from the accumulation of misfolded proteins, caused by oxidative stress. Third, there appears to be shift in metabolic and detoxification pathways as these are elevated in both HT-29 and HCT116. This would suggest differential regulation of detoxification genes, possibly due to exposure to ER or its metabolites. Finally, changes in cholesterol homoeostasis pathway suggest changes in lipid metabolism or membrane composition. These are further represented in a heatmap (Figure 6).

**FIGURE 7:**
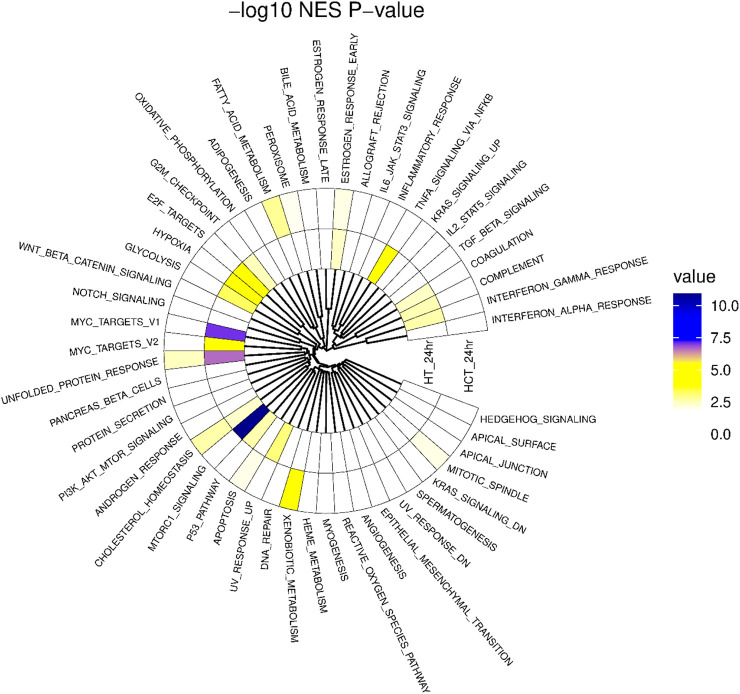
Landscape Plot depicting the -log10p-values of each hallmark (n = 50) pathway across the four exposure groups. Pathways are ordered in the circular view based on hierarchal clustering of the gene membership (i.e., gene set similarity).

A landscape plot to was constructed to visualize important ferroptotic pathways influenced by ER treatment in both HT-29 and HCT116 cells at 24 h. As shown in Figure 7, several biologically relevant pathways common to both cell lines were affected by ER treatment. Pathways that relate to and define ferroptosis are summarized in Table 1. We found that ROS and unfolded protein response (UPR) pathways were significantly enhanced in both cell lines while M-TORC1 Signaling and Fatty Acid Metabolism were decreased. MTORC1 signaling is indicative of enhanced ferroptosis as MTORC1 controls both lipid metabolism (Cheon and Cho, 2021) and cholesterol synthesis (Lu et al., 2020).

**TABLE 1:** Comparative table for ferroptosis-related Hallmark pathways common to both HT-29 and HCT116 cells following Erastin treatment for 24 h. NES, Normalized Enrichment Score.

Pathway	HT-29 NES	HCT116 NES	Significance
Interferon alpha response	0.74	2.47	Strong immune activation
Interferon gamma response	4.26	1.20	Strong immune activation
Glycolysis	5.03	1.85	Metabolism shift, enhanced in HT-29
ROS pathway	0.51	2.41	Increased ROS in HCT116
Unfolded protein response (UPR)	9.45	3.97	Oxidative stress-induced protein folding
Xenobiotic metabolism	3.18	5.22	Detoxification of reactive species
Oxidative phosphorylation	1.06	1.13	Decreased in both, consistent with ferroptosis
M-TORC1 signaling	12.26	2.34	Higher activity in HT-29 than HCT116
Fatty acid metabolism	1.47	4.34	Increase in lipid metabolism, more in HCT116

### RT-PCR studies

**FIGURE 8:**
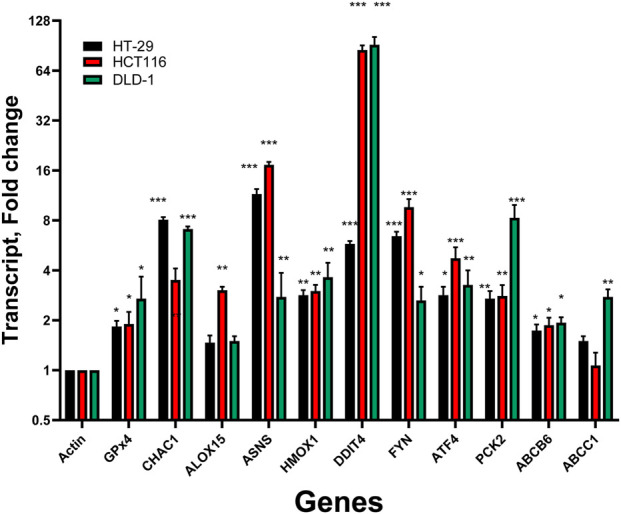
RT-PCR results for colon cancer cells following 24 h of Erastin treatment. RNA was isolated, purified, and subjected to RT-PCR as described in the Methods section. Gene expression was normalized to β-actin, and expression levels in each treatment group were compared with those of time-matched controls. Data are presented as fold change relative to β-actin. *, **, and *** indicate p < 0.05, p < 0.005, and p < 0.001, respectively.

RT-PCR studies were carried out to confirm ER induced DETs that were found to be common in both cell lines by microarray analysis and have been implicated in the process of ferroptosis. To expand our comparisons, we also included DLD-1, another KRAS-mutant cell line representing the more “classical” adenoma–carcinoma pathway. Thus, HCT116, HT-29, and DLD-1 complement each other, reflecting a spectrum of genetic backgrounds frequently used in CRC research. The results of RT-PCR are shown in Figure 8.

Several common DETs observed in Microarray analysis of CRC cells following ER treatment (Supplemetary Table 2) were confirmed by RT-PCR (Figure 8). *CHAC1* has been found to be an important gene for oxidative stress (Sun et al., 2024) and ferroptosis (Sinha et al., 2023). We have previously observed significant induction of CHAC1 in CRC during ferroptosis by NCX4040 as well as in other tumor cells undergoing ferroptosis (Sinha et al., 2023). Heme oxygenase1, a biomarker gene for oxidative stress (Liu et al., 2011; Yue et al., 2015), was universally induced in all CRC cell line we tested (Supplementary Table 3) at 24 h, suggesting a strong ER-induced oxidative stress in these cells. Glutathione Peroxidase 4 (*GPX4*), an important gene for modulating ferroptosis, was slightly induced. It has been suggested that this upregulation of *GPX4,* in some cancer cells, is a compensatory mechanism to respond to ER-induced stress to prevent death from ferroptosis.

ER treatment slightly increased expressions of ALOX5 gene which encodes for arachidonate15-lipoxygenase (also known as 15-LOX) is known to be involved in generating lipid mediators that regulate inflammation and other biological processes. ALOX15 is an important meditator of ferroptosis as it catalyzes the oxidation of polyunsaturated fatty acids (PUFAs) in cell membranes, leading to the production of ROS and lipid hydroperoxides, a hallmark of ferroptosis.

ASNA (Asparagine synthetase) is a gene that encodes for enzyme asparagine synthetase to produce aspartate and glutamine, an essential amino acid for protein synthesis. ASNS have been reported to be involved in ferroptosis and is now considered a biomarker gene in CRC, hepatocarcinoma and prostate cancer (Chen et al., 2021a; Gan et al., 2024) along with PCK2 gene (Qi et al., 2025; Li et al., 2025). Our RT-PCR results showed that ASNS gene was highly induced by ER in both HT-29 and HCT116, however, less in DLD-1 cells.

Our results also show that *DDIT4* (DNA damage-inducible transcript 4) was upregulated in HT-29 cells (5.5-fold) while it was highly induced in both HCT116 and DLD-1 cells. DDIT4 is a stress-related gene that is upregulated in response to DNA damage, hypoxia, and endoplasmic reticulum damage. DDIT4 encodes a protein that has been reported to inhibit mTORC1 activity, a signaling pathway involved in cell growth, metabolism, and survival, resulting in cytotoxicity under stress conditions, especially under oxidative stress. Upregulation of DDIT4 gene has been shown to enhance ferroptotic cell death in lung cancer and in neurons (Zhou et al., 2021; Luo et al., 2023).

RT-PCR indicated ER treatment caused significant upregulations of FYN gene in both HT-29 and HCT116 cells, and a smaller upregulation in DLD-1 cells. The FYN gene encodes Fyn tyrosine kinase, a member of the Src family of kinases which is involved in cell growth, adhesion, and signaling. While Fyn is not directly involved in ferroptosis, Fyn can activate CLDN6 through phosphorylation, promoting NrF2 nuclear export and inducing ferroptosis (Qi et al., 2025). ER treatment also caused an upregulation of ATF4 (Activating transcription factor 4) in all three CRC cells. ATF4, a mediator of metabolic, oxidative homeostasis and cell survival, is upregulated in response to diverse microenvironmental stresses, ER stress damage, and oxidative stress (Kress et al., 2023; Tang et al., 2024). The function of ATF4 in ferroptosis is complex: it can act both as an inhibitor or it can upregulate ferroptosis (Chen et al., 2017; Tang et al., 2024).

ABC transporters, especially ABCB6 and ABCC1, have been implicated in ferroptosis (Zhang et al., 2025; Chen et al., 2021b; Yang et al., 2025). ABCC1 is involved in the efflux of GSH, making cells more susceptible to ferroptosis. High ABCC1 expression in tumor cells can thus make cells more sensitive to ferroptosis-inducing agents, like Erastin, due to the GSH depletion caused by ABCC1’s activity. Our RT-PCR studies showed that ABCB6 was slightly upregulated by ER treatments while ABCC1 expression was only increased in DLD-1 cells.

## Discussion

Colon cancer is one of leading causes of death worldwide due to failure to treatment, resulting from the development of chemotherapy resistance. Unfortunately, there are not many CRC-selective, devoid of resistance, drugs available. There is significant need for better drugs or combinations of drugs for treatment of CRC. Ferroptosis, a new form of cell death, is characterized by iron-dependent lipid peroxidation, and has gained a great deal of recognition as a promising therapeutic avenue. Human CRC contain RAS mutations, most commonly at G13D and ferroptosis inducers, such as Erastin or RSL3, are effective against tumors harboring KRAS mutations. Both Erastin and RSL3 induce ferroptosis by inhibiting functions of GPX4, leading to generation of ROS (and lipid ROS) and that ultimately result in extensive cell membrane peroxidation and death. In recent years, combinations of ER and RSL3 with other chemotherapeutic agents have been shown to highly effective against several difficult to cure human cancers, including ABC-overexpressing tumor cells (Zhou et al., 2019; Perera et al., 2025). We have shown that combinations of ER or RSL3 with Doxorubicin or Topotecan were extremely effective in reversing MDR in both ABCB1 and ABCG2-expressing cell lines (Perera et al., 2025). Furthermore, combinations of ER with Taxol were shown to be effective in reversing MDR in a ABCB1-expressing tumor cell line (Zhou et al., 2019). These observations suggest that ferroptosis inducers may be extremely important in combinations with current drugs to treat human cancers.

However, several critical issues remain unresolved. Most importantly, there is the lack of a clear understanding of the mechanisms and pathways involved in ferroptosis. Additionally, different inducers have different mechanisms of action that are tissue and cell dependent. Early research has identified that Glutathione Peroxidase 4 (*GPX4*), an antioxidant enzyme that utilizes GSH as a cofactor to catalyze the reduction of lipid peroxides, is a key regulator of ferroptosis. Inhibition of Solute Carrier Family 7 Member 11 (*SLC7A11*) leads to the reduction of GSH, and increase in ROS formation, triggering oxidative damage and ferroptosis. The *SLC7A11*-GSH-*GPX4* pathway is recognized as the classical pathway of ferroptosis.

Inducing or promoting cellular ferroptosis with ER or RSL3 (or their combinations with various other anticancer drugs) is a promising tumor therapy option in the clinic. However, the role of ferroptosis in CRC is not fully elucidated. Furthermore, identification of ferroptosis-related pathways, metabolic and prognostic genetic biomarkers with clinical significance in CRC have not been comprehensively evaluated. In this study, we have explored the differential expression of ferroptosis-related genes in CRC-related HT-29 and HCT116 cells in response to ER. Despite its limited clinical utility due to suboptimal pharmacokinetic properties, ER remains a valuable experimental probe for elucidating the molecular mechanisms and cellular biology of ferroptosis. Here, we have utilized Gene Set Enrichment Analysis (GSEA) to explore functionally related pathways to identify potential biomarkers genes and therapeutic targets for early diagnosis and prognosis of CRC, leading to improved clinical strategies. Biomarkers are important tools for early detection in the treatment of cancers with chemotherapy. Biomarkers, including genes, offer many advantages in chemotherapy, helping in selection of personalized cancer treatment, and improved treatment outcomes with reduced risks. Furthermore, the identification of biomarkers of ferroptosis can significantly enhance the field of chemotherapy by providing tools to monitor, predict, and potentially mitigate cell death of normal cells/tissues due to iron-dependent lipid peroxidation.

Our studies show that some transcripts were highly enriched in both HT-29 and HCT-116 cell line following ER treatment at 24 h. Since few transcripts or pathways were observed at 4 h in these cell lines, most of our analysis with differential gene expressions and pathways were carried out at 24 h. While many transcripts were induced in HT-29 and HCT116 cells by ER, 26 transcripts were found to be common in both cell line (Table 1). Several of the common transcripts (e.g., ASNS, PCK2, CHAC1, and DDTI4) were highly enriched in both cell lines, indicating a common, fundamental response to ER treatment leading to either processes for cell survival or to ER-dependent ferroptotic cell death. Several of these transcripts have been previously shown to be upregulated during the process of ferroptosis, however, the expression these genes have not been observed in colon cell lines. Our RT-PCR studies conducted with HT-29, HCT116 and DLD-1 cells also confirmed the induction of these genes following treatment with ER. Our study suggests that ASNS, CHAC1, PCK2, DDIT4 and ATF3/4 may represent biomarker genes for CRC and other human tumors undergoing ferroptosis.

In this study we have also identified several common pathways that were significantly affected by ER at 24 h (Supplementary Table 4; Figure 5). Notable pathways that were influenced by ER were EIF2AK1-Heme deficiency, unfolded protein response (UPR) and changes in xenobiotic metabolism, indicating differential regulation of detoxification genes, possibly due to exposure to ER or its metabolites. Heme catabolism and UPR are well known oxidative stress responses, leading to ferroptosis. A Landscape plot (Figure 7) also identified important ferroptosis-related pathways that were differently modulated by ER in HT-29 and HCT116 cells. Interferon-dependent immune response was enriched in HT-29 by ER (HALLMARK_INTERFERON_GAMMA_RESPONSE NES = 4.26, adjusted p-value = 0.0115) In HT-29, four of the 5 DETs in this pathway were upregulated. While Hallmark interferon-dependent immune response pathways were not significantly enriched in HCT116, the alpha and gamma response pathways each contained three upregulated DETs.

Our studies also show that ER treatment significantly enriched glycolysis in HT-29 (HALLMARK_GLYCOLYSIS pathway NES = 5.03, adjusted p-value = 0.0034). Four of 6 DETs in this pathway in HT-29 were upregulated. This pathway was not significantly enriched in HCT116 cells (NES = 1.85, adjusted p-value = 0.2216). However, all 4 DETs in this pathway were upregulated. Upregulation of glycolysis concomitantly decreases oxidative phosphorylation, an effect known as the Warburg effect (Vander Heiden et al., 2009; Papaneophytou, 2024). This metabolic rewiring would suggest that ER caused a significant metabolic shift in these cells to produce ATP through glycolysis to compensate for increased cellular oxidative stress to survive ER-induced ferroptosis (Gotorbe et al., 2022).

In this study several pathways were identified, e.g., glycolysis, ROS, UPR, xenobiotic metabolism which are highly relevant to the mechanism of ER-induced ferroptosis. However, it should be noted that their magnitude and mode of activation depend on the inducer’s mechanism—whether upstream (e.g., Xc^−^ inhibition by Erastin) or downstream (e.g., GPX4 inhibition by inducers). These findings also suggest therapeutic opportunities. Since many cancers already harbor elevated ROS, further increasing ROS via ferroptosis inducers could selectively kill tumor cells while sparing normal tissue. This could be enhanced by combining ferroptosis inducers with ROS-generating therapies, e.g., radiation, doxorubicin, photodynamic therapy (Perera et al., 2025; Ye et al., 2020; Lei et al., 2021) while simultaneously blocking antioxidant defenses (NRF2, GSH, thioredoxin).

Erastin-induced glycolysis represents a survival adaptation, rendering tumor cells more dependent on glucose metabolism (Yao et al., 2021; Kirkwood-Donelson et al., 2024). This vulnerability could be exploited by combining ferroptosis inducers with glycolysis inhibitors (e.g., 2-deoxyglucose) or pentose phosphate pathway (PPP) inhibitors, thereby blocking NADPH generation and enhancing tumor killing. UPR pathways were also upregulated in both cells indicating endoplasmic reticulum stress, due to accumulation of misfolded proteins, resulting from oxidative stress, central to ER mechanism of ferroptosis. Xenobiotics pathways identified in this study is a novel finding and has not been previously reported in CRC with ER. This would suggest a shift in metabolism and detoxification of ER as the pathway was found to be elevated in both HT-29 and HCT116. This is currently under investigation as this may have implications in ER resistance in HCT116 cells.

While mechanisms of ER sensitivity were not the focus of this study, differential sensitivity of ER in CRC cell lines have been noted before (Gotorbe et al., 2022; Hu et al., 2022). However, the underlying mechanism of resistance is not known. Hence, elucidating the mechanism of resistance of colon cancer cells to Erastin-induced ferroptosis are of utmost importance.

Our studies, however, indicated that ROS metabolic activity was slightly enhanced in HCT116 cells by ER treatment, as evidenced in the gene enrichment analysis. *SOD1* was significantly upregulated in the HCT116 cell line, suggesting detoxication of O_2_^−.^ (superoxide anion radical) and ROS, thereby decreasing lipid peroxidation, causing a decrease in ER-dependent ferrototic cell death. In addition, gene enrichment analysis showed that expression of *NQO1 (NAD(P)H quinone dehydrogenase 1)* was also elevated in the HCT116 cell line, suggesting further detoxification of ER-induced ROS and its metabolites in cells as *NQO1* inhibits free radical formation and protects cells from oxidative stress (Luo et al., 2018; Ma et al., 2024). *NQO1* has also been shown to maintain the reduced forms of endogenous antioxidants (e.g., ubiquinone and alpha-tocopherol), which are crucial for protecting cellular membranes from peroxidation (Ergen et al., 2007; Ross and Siegel, 2017). These events, e.g., increased *SOD1* and *NQO1* expression would result in enhanced detoxification of ER-dependent ROS, leading to a decreased ER sensitivity to HCT116 cells as observed here. Xenobiotic metabolism pathways were also enriched in HCT116 cells (8 DET’s, six upregulated and two downregulated, NES 5.2) compared to HT-29 cells (4 DET’s, two upregulated and two downregulated, NES 3.18), suggesting an enhanced detoxification of reactive metabolites of ER in HCT116 cell line. Work is in progress to address these in detail and to further evaluate roles of these genes in ER sensitivity in CRC cells and will be reported separately at a future time.

In conclusion, our study has identified several genes in CRC cells that are characteristics of ferroptosis induced by Erastin, and 15 transcripts were found to be common in both cell lines at the more stringent foldchange threshold of 1.5. Several of these genes (e.g., *ASNS, PCK2, CHAC1*, and *DDTI4*) were highly enriched in both cell lines, indicating a common response to ER-induced cell death. Induction of these genes was confirmed by RT-PCR in three CRC cell lines; HT-29, HCT116 and DLD-1. Our findings indicate that *ASNS, CHAC1, PCK2 DDIT4* along with *ATF3/4* may represent major biomarker genes for CRC. Monitoring these ferroptosis-related gene biomarkers may be helpful in identifying patients who will respond to ferroptosis inducers and develop personalize treatment options.

## Data Availability

The datasets presented in this study can be found in online repositories. The names of the repository/repositories and accession number(s) can be found in the article/Supplementary Material.
