# Supplementary material for: Gene expression profiling identifies ferroptosis-related genes and pathways in human colon cancers cell lines
Source: Front Mol Biosci. 2026 Jan 20;12:1680206. doi: 10.3389/fmolb.2025.1680206 (PMC12865207; doi:10.3389/fmolb.2025.1680206)

**Supplementary File**

Supplementary Table-1: Primers used and their sequences

**Beta-Actin**: CACCATTGGCAATGAGCGGTTC-forward; AGGTCTTTGCGGATGTCCACGT-reverse

**GPX4**: ACAAGAACGGCTGCGTGGTGAA-forward; GCCACACACTTGTGGAGCTAGA-reverse

**CHAC1**: GTGGTGACGCTCCTTGAAGATC-forward; GAAGGTGACCTCCTTGGTATCG-reverse

**HMOX1**: CCAGGCAGAGAATGCTGAGTTC-forward; AAGACTGGGCTCTCCTTGTTGC-reverse

**ALOX15**: ACCTTCCTGCTCGCCTAGTGTT-Forward; GGCTACAGAGAATGACGTTGGC-Reverse

**ASNS**: CTGTGAAGAACAACCTCAGGATC-Forward; AACAGAGTGGCAGCAACCAAGC-Reverse

**DDIT4**: GTTTGACCGCTCCACGAGCCT-Forward; GCACACAAGTGTTCATCCTCAGG-Reverse

**FYN**: CTGGTCACCAAAGGAAGAGTGC-Forward; GGTCCTTTTTCCAGCAGTGGATC-Reverse

**ATF4**: TTCTCCAGCGACAAGGCTAAGG-Forward; TTCTCCAGCGACAAGGCTAAGG-Reverse

**ABCB6**: GTTCTTCAACGCCTGGTTTGGC-Forward; AGCACGACGAAACTTGGTTCTCC-Reverse

**ABCC1**: CCGTGTACTCCAACGCTGACAT-Forward; ATGCTGTGCGTGACCAAGATCC-Reverse

**SLC7A11**: TCCTGCTTTGGCTCCATGAACG-Forward; AGAGGAGTGTGCTTGCGGACAT-Reverse

**PCK2**: TAGTGCCTGTGGCAAGACCAAC-Forward; GAAGCCGTTCTCAGGGTTGATG-Reverse

**Supplementary Table-2**: summary of the 15 common DETs at 24hr in HT-29 and HCT116 cell lines with fold-change ≥ 1.5.


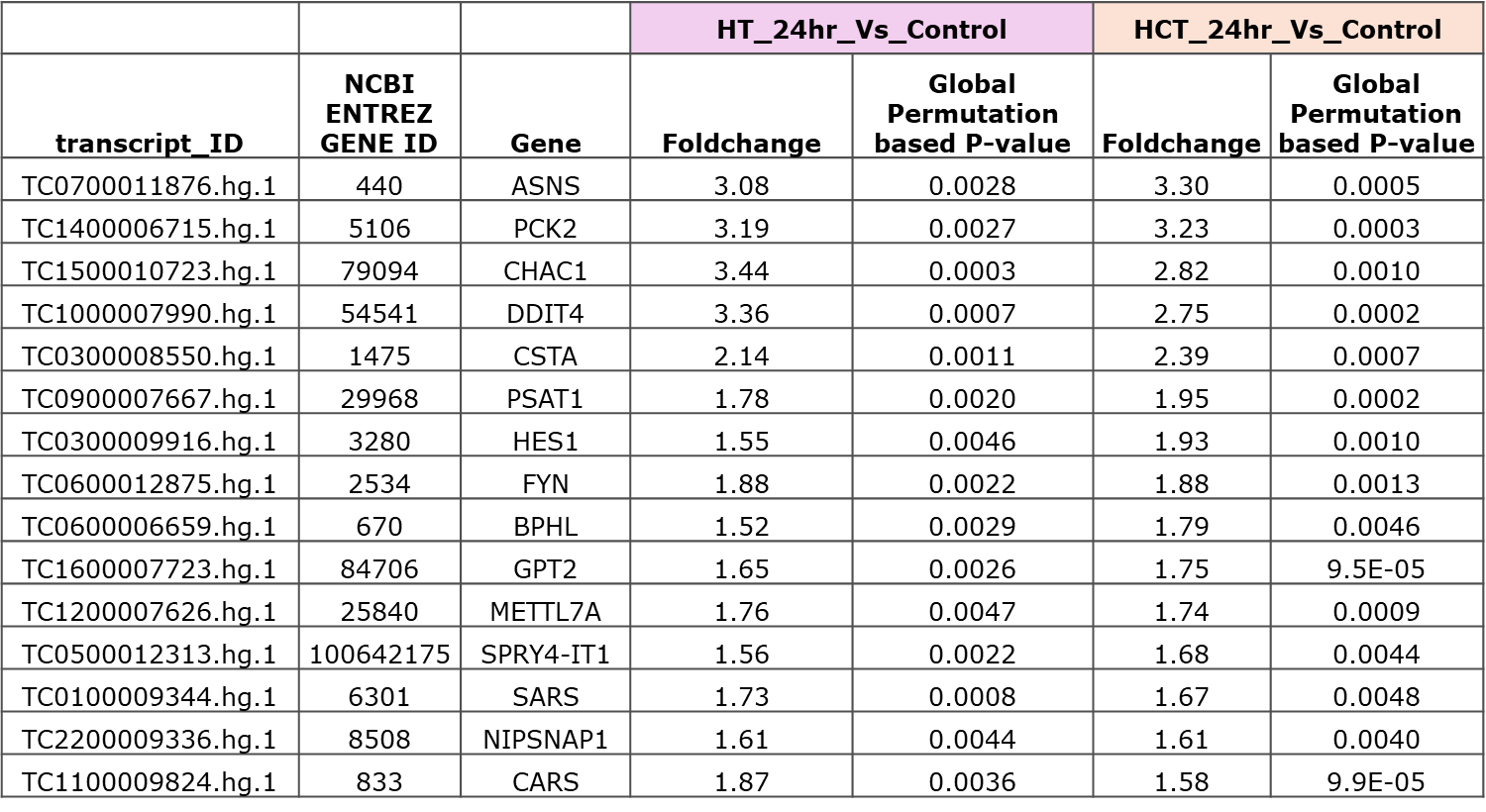


**Supplementary Table-3**: Differentially enriched pathway (DEPs) for HT-29 and HCT116 cells.

| **Cell Line** | **Time**  **(vs. Control)** | **# DEPs** |
| --- | --- | --- |
| HT | 4hr | 1 |
| HT | 24hr | 61 |
| HCT | 4hr | 0 |
| HCT | 24hr | 16 |

**Supplementary Table-4**: Common differentially enriched pathways (DEPs) at 24 h in HT-29 and HCT116 cells


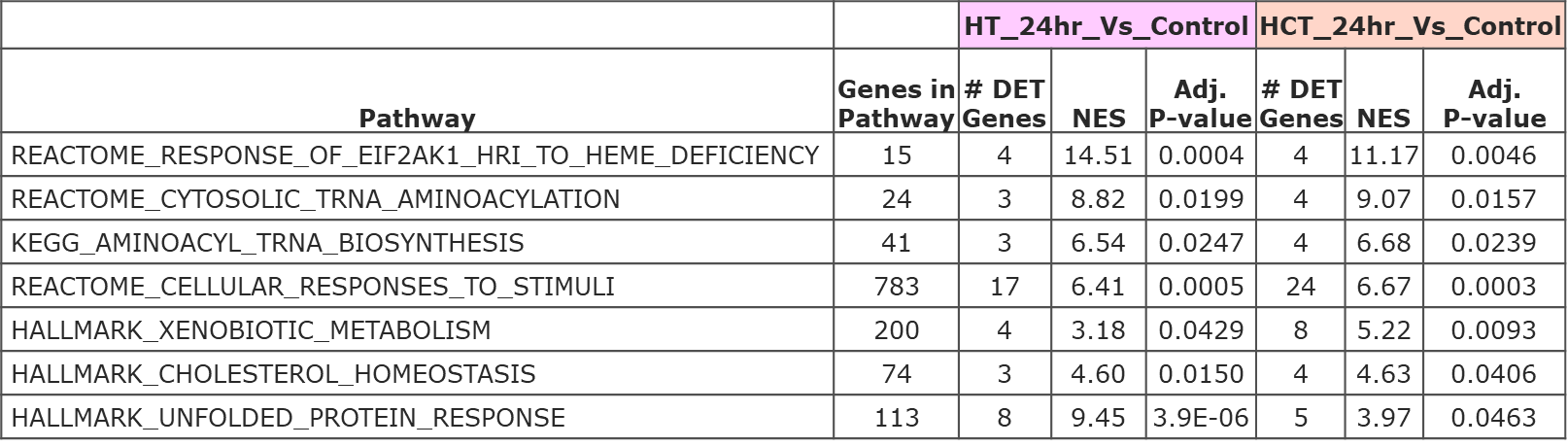

Supplement: Supplementary file 1 [file Supplementaryfile1.docx]
